# Application of Soft Computing Represented by Regression Machine Learning Model and Artificial Lemming Algorithm in Predictions for Hydrogen Storage in Metal-Organic Frameworks

**DOI:** 10.3390/ma18133122

**Published:** 2025-07-01

**Authors:** Jiamin Zhang, Yanzhe Li, Chuanqi Li, Xiancheng Mei, Jian Zhou

**Affiliations:** 1 SINOPEC Research Institute of Petroleum Engineering, Beijing 100101, China; zhangjm48274.sripe@sinopec.com; 2 School of Intelligent Software and Engineering, Nanjing University, Suzhou 215163, China; yanzhe.lee@smail.nju.edu.cn; 3 School of Resources and Safety Engineering, Central South University, Changsha 410083, China; j.zhou@csu.edu.cn; 4 State Key Laboratory of Geomechanics and Geotechnical Engineering, Institute of Rock and Soil Mechanics, Chinese Academy of Sciences, Wuhan 430071, China

**Keywords:** hydrogen storage, metal-organic frameworks, machine learning, artificial lemming algorithm, prediction

## Abstract

Metal-organic frameworks (MOFs) have been extensively studied for hydrogen storage due to their unique properties. This paper aims to develop several regression-based machine learning models to predict the hydrogen storage capacity of MOFs, including artificial neuron network (ANN), support vector regression (SVR), random forest (RF), extreme learning machine (ELM), kernel extreme learning machine (KELM), and generalized regression neural network (GRNN). An improved population-based metaheuristic optimization algorithm, the artificial lemming algorithm (ALA), is employed to select the hyperparameters of these machine learning models, enhancing their performance. All developed models are trained and tested using experimental data from multiple studies. The performance of the models is evaluated using various statistical metrics, complemented by regression plots, error analysis, and Taylor graphs to further identify the most effective predictive model. The results show that the ALA-RF model obtains the best performance in predicting hydrogen storage, with optimal values of coefficient of determination (R^2^), root mean square error (RMSE), Willmott’s index (WI), and weighted average percentage error (WAPE) in both training and testing phases (0.9845 and 0.9840, 0.2719 and 0.2828, 0.9961 and 0.9959, and 0.0667 and 0.0714, respectively). Additionally, pressure is identified as the most significant feature for predicting hydrogen storage in MOFs. These findings provide an intelligent solution for the selection of MOFs and optimization of operational conditions in hydrogen storage processes.

## 1. Introduction

The accelerating global energy crisis, driven by the depletion of fossil fuels and growing pressure to achieve carbon neutrality, has led to a search for sustainable, high energy density solutions [1]. Hydrogen energy stands out as a promising alternative due to its zero-emission profile and high gravimetric energy density, though its practical application is hampered by low volumetric densities and demanding storage requirements such as high-pressure or extremely low-temperature conditions [2].

Among various hydrogen storage media, metal-organic frameworks (MOFs), crystalline structures composed of metal nodes and organic linkers, have attracted strong research interest [3]. Their exceptional surface area, tunable pore geometry, and modular chemical structure enable highly selective and reversible hydrogen adsorption under moderate conditions [4]. These properties make MOFs ideal candidates for physisorption-based hydrogen storage under moderate conditions [5].

Conventional approaches to optimize MOF structures, whether via high-throughput simulations or empirical synthesis, are often costly, time-consuming, and sensitive to experimental conditions, limiting their rapid discovery and scale-up [6]. For instance, Avci et al. [7] performed an extensive computational screening of MOF adsorbents and membranes for CO_2_/H_2_ separation, uncovering H_2_/CO_2_ selectivity ranging from 2.1 × 10^−5^ to 6.3 and H_2_ permeabilities between 230 and 1.7 × 10^6^ Barrer under pressure swing adsorption (PSA) and vacuum swing adsorption (VSA) conditions; yet each simulation run required over 10^4^ CPU hours, and key effects such as charge transfer at open metal sites were undervalued due to simplified force-field models. Meanwhile, empirical synthesis and characterization of MOFs often follow iterative, trial-and-error protocols. According to the EU HyStorPor project, developing a novel MOF for hydrogen storage averages 18 months and costs approximately EUR 200,000, underscoring the impracticality of exhaustive laboratory screening [8]. The performance of MOFs is highly sensitive to subtle environmental fluctuations [9]. Minor temperature shifts can alter coordination bonds, modifying pore size and adsorption sites, while pressure variations affect crystal packing density and accessible surface area [10]. These dynamic responses necessitate repeated experiments or simulations under finely controlled conditions, further prolonging the discovery cycle.

Machine learning (ML) has emerged as a powerful tool to predict material properties by uncovering complex, non-linear relationships within existing datasets [11,12,13,14,15]. Yuan et al. demonstrated the potential of tree-based models by training gradient boosting decision trees (GBDTs) on a diverse dataset of porous carbons, achieving an R^2^ of 0.98 on the training set and 0.84 on held-out test data, demonstrating a strong fit and good generalization ability of the model [16]. They further applied the GBDT classifier to distinguish between regular porous carbon (RPC) and heteroatom-doped porous carbon (HDPC), where the model again excelled. On this basis, Wang et al. [17] conducted a hierarchical screening of approximately 330,000 hypothetical MOFs for methane adsorption, ultimately identifying the four best candidates, which had a calculated working capacity of 145 cm^3^/cm^3^ at 5–35 bar and 298 K, close to the U.S. Department of Energy’s 2015 target of 180 cm^3^/cm^3^.

In addition to tree-based approaches, further advances have been made in neural networks and hybrid algorithms. Anderson et al. [18] trained a multilayer perceptron (MLP) to predict complete adsorption isotherms, finding that the MLP’s outputs correlated linearly with Grand Canonical Monte Carlo (GCMC) simulations when loading exceeded 50% of saturation, while maintaining clustering near the parity line at low loadings (<25%). Wei et al. [19] combined random forest (RF) with k-nearest neighbors (KNN) to handle high-dimensional synthesis data, revealing key features that generalize across MOF chemistries. Guo et al. [20] compared MLP and long short-term memory (LSTM) networks for gas adsorption prediction, showing that deep learning models outperformed conventional RF methods, especially under low-pressure conditions.

A solid foundation for ML-driven MOF property prediction has been established based on previous studies, yet two critical challenges remain. On the one hand, most studies evaluate either one or more ML techniques in isolation without exploring the relative merits of other regressors. Pardakhti et al. [21] trained a random forest on just 8% of 130,398 hypothetical MOFs and achieved an R^2^ of 0.98 (training) and a mean absolute percent error of ~7% (testing), completing all predictions in roughly two hours on a single desktop, which was orders of magnitude faster than comparable molecular simulations. To further accelerate screening, Choudhary et al. [22] applied the Atomistic Line Graph Neural Network (ALIGNN) to predict CO_2_ adsorption across 137,953 hypothetical MOFs, demonstrating orders-of-magnitude speedups over Grand Canonical Monte Carlo simulations while retaining high accuracy. On the other hand, hyperparameter tuning remains a key issue: grid searches can only exhaustively sample a very small fraction of the combined space, typically less than 0.1% of the possible configurations, leading to high computational costs in high dimensions [23,24]. Bayesian optimization addresses this by modeling performance as a Gaussian process and sequentially selecting promising hyperparameter sets, routinely outperforming grid and random search and matching or exceeding expert-tuned results [25,26]. Building on this, metaheuristic methods transfer knowledge from prior tuning tasks to new problems, slashing hyperparameter optimization times from days to under two hours by leveraging surrogate performance predictors trained on historical trials, but such strategies have yet to be systematically applied to MOF discovery [27].

To bridge the identified research gaps, this study introduces an innovative integrated framework that synergizes six regression ML models with metaheuristic optimization algorithms, with the aim of achieving three overarching objectives: (1) compare the predictive accuracy of six ML models, including artificial neuron network (ANN), support vector regression (SVR), random forest (RF), extreme learning machine (ELM), kernel extreme learning machine (KELM), and generalized regression neural network (GRNN), in estimating the hydrogen storage capacities of MOFs; (2) embed a metaheuristic algorithm called artificial lemming algorithm (ALA) to improve hyperparameter optimization, ensuring more rapid convergence and enhanced model generalizability across different datasets; and (3) conduct comprehensive sensitivity analysis to pinpoint the key physicochemical features that govern hydrogen storage in MOFs. The remainder of this paper is structured as follows: Section 2 provides an in-depth description of the methodologies employed, including details on the ML models, the enhanced metaheuristic optimization algorithm, and the sensitivity analysis approach. Section 3 delves into the characteristics and composition of the database used in this paper. Section 4 outlines the development and implementation of prediction models. Section 5 presents and discusses the results obtained from comparative analysis, hyperparameter optimization, and model interpretability. Finally, Section 6 summarizes the key findings, highlights the study’s contributions, and outlines directions for future research.

## 2. Methodologies

### 2.1. Artificial Neuron Network (ANN)

**Figure 1:**
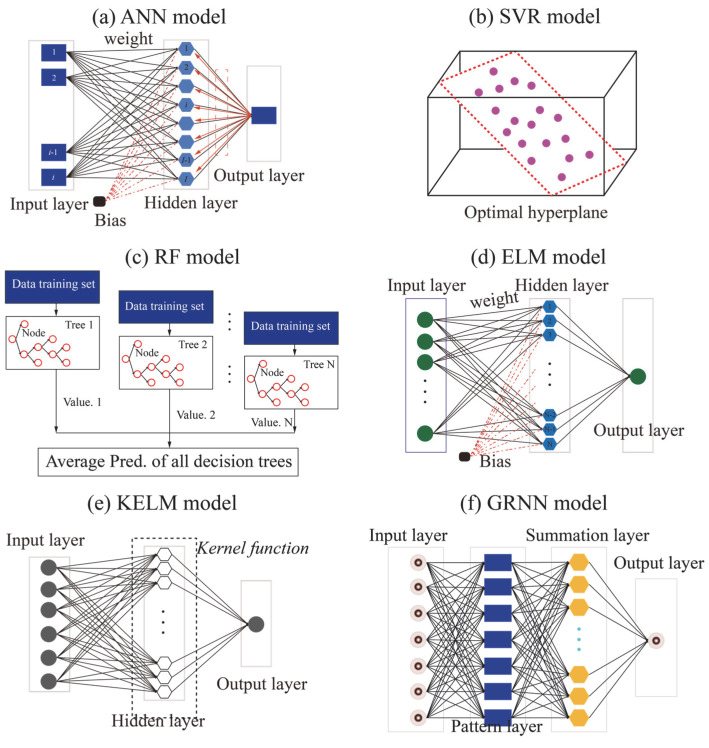
Demonstration of all ML models used in this paper.

ANNs are a class of computational models inspired by biological neural systems, designed to learn and perform complex tasks in pattern recognition, classification, and regression [28]. An ANN typically comprises multiple layers of neurons (also referred to as nodes), which interact through weighted connections. As illustrated in Figure 1a, a standard ANN architecture comprises an input layer, one or more hidden layers, and an output layer. The input layer receives external data. Composed of numerous neurons, the hidden layers perform feature extraction and nonlinear transformation of the input. The output layer produces the final output of the network, which is commonly for classification or regression tasks. In the context of regression prediction using an ANN model, the model output can be mathematically represented as follows:

(1) $$y=f\left(\sum_{i=1}^{n}w_{i}x_{i}+b\right)$$

where *x* and *y* denote the input and output features, respectively. *w_i_* represents the weight of the *i*-th feature, and b represents a bias value. *f*() is an activation function.

### 2.2. Support Vector Regression (SVR)

Support vector machines (SVMs) are a type of supervised learning method applicable to both classification and regression tasks. The core concept of SVMs is to identify an optimal hyperplane that maximally separates samples of different classes, thereby enhancing the model’s generalization ability. Originally proposed by Vapnik et al. [29] and grounded in statistical learning theory, SVMs possess a solid mathematical foundation. The primary objective is to find a hyperplane in the feature space that not only correctly classifies the data points but also maximizes the margin between different classes. For regression tasks, the support vector regression (SVR) model adapts the SVM framework by introducing an *ε*-insensitive loss function, making it suitable for predicting continuous outcomes. The goal in SVR is to identify an optimal hyperplane that fits the data within a specified tolerance level of error (see Figure 1b). To this end, SVR typically employs an *ε*-insensitive loss function and constraint conditions to formulate the prediction objective equation.

(2) $$L(y,f\left(x\right))=\left\{\begin{array}{c} 0\\ \left|y-f\left(x\right)\right|-\varepsilon \end{array}\right.\begin{array}{c} \left|y-f\left(x\right)\right|\leq \varepsilon \\ \left|y-f\left(x\right)\right|>\varepsilon \end{array}$$

(3) $$\underset{w,b}{\min}\frac{1}{2}{\left\|w\right\|}^{2}+C\sum_{i=1}^{n}\left(\xi_{i}+\xi_{i}^{*}\right)$$

where $\left\|w\right\|$ is the regularization coefficient that controls model complexity. *C* is a penalty parameter. $\xi_{i}$ and $\xi_{i}^{*}$ are slack variables introduced to allow certain deviations from the ε-insensitive tube. To enhance the model’s applicability to nonlinear data, the kernel method is employed to map the original data into a higher-dimensional feature space, where linear separation becomes feasible. In this paper, the radial basis function (RBF) kernel is selected to further improve the predictive performance of the SVR model.

### 2.3. Random Forest (RF)

RF is an ensemble learning method widely used for classification and regression tasks. By introducing randomness into the decision tree (DT) construction process, RF improves both the model’s generalization ability and robustness. RF regression is a type of bootstrap aggregation (bagging) technique that builds multitude decision trees and averages their outputs to produce the final prediction. This ensemble approach effectively reduces overfitting while improving model stability and accuracy [30]. The RF training process can be summarized in Figure 1c and involves the following steps: (1) random sampling of training data (with replacement); (2) random sampling of features at each node split; (3) construction of individual DTs; and (4) aggregation of multiple DTs. For regression tasks, the final prediction is obtained by averaging the outputs of all the individual DTs:

(4) $$y=\frac{1}{N}\sum_{n=1}^{N}DT_{n}$$

where *N* represents the maximum number of DTs in an RF model.

### 2.4. Extreme Learning Machine (ELM)

ELM is a single-hidden-layer feedforward neural network characterized by high computational efficiency. In the ELM model, the weights and biases of the hidden layer neurons are randomly assigned and remain fixed, while the output weights are analytically determined using the least squares method. This approach enables fast learning and training, distinguishing ELM from traditional neural network training methods such as backpropagation, which require iterative updates of weights and biases. By eliminating the need for backpropagation, ELM significantly simplifies the training process and greatly improves training speed [31]. As shown in Figure 1d, the ELM architecture typically consists of three layers: (1) an input layer receives external data; (2) a single hidden layer comprising multiple neurons that perform nonlinear transformations of the input. The weights and biases in this layer are randomly initialized and require no training; and (3) an output layer produces the final output of the network.

### 2.5. Kernel Extreme Learning Machine (KELM)

KELM is an advanced version of ELM that incorporates kernel methods to better handle nonlinear problems. As shown in Figure 1e, KELM bypasses explicit hidden layer construction by using a kernel function to map input data into a high-dimensional feature space. This allows the model to capture complex patterns more effectively. The output weights are computed analytically through a regularized least squares solution, thus retaining ELM’s fast learning speed and strong generalization performance. In this paper, the RBF kernel is also employed to further improve the predictive performance of the KELM model.

### 2.6. Generalized Regression Neural Network (GRNN)

GRNN is a type of single-hidden-layer feedforward neural network based on kernel density estimation and conditional expectation theory. GRNN assumes that the input and output variables follow a joint probability distribution. The relationship between inputs and output is modeled using conditional expectation. It estimates the joint probability density function from training samples and computes the conditional expectation to generate predictions. GRNN consists of four layers: an input layer, a pattern layer, a summation layer, and an output layer (see Figure 1f). This structure enables GRNN to achieve fast convergence and high accuracy, particularly in nonlinear regression problems [32].

### 2.7. Artificial Lemming Algorithm (ALA)

Xiao et al. [33] proposed a novel metaheuristic algorithm, called the Artificial Lemming Algorithm (ALA), designed to address common challenges in high-dimensional search spaces, such as premature convergence, insufficient exploration, and lack of robustness. The algorithm is inspired by the natural behaviors of lemmings, including long-distance migration, burrowing, foraging, and evading predators. In the wild, lemmings exhibit a unique behavioral pattern that provides a rich biological basis for algorithm design: (1) Long-distance migration–This behavior often occurs when population density surpasses environmental carrying capacity and food resources becomes limited. Rather than being a deliberate search for new habitats, migration represents a spontaneous group-level response to ecological stress. As the population increases, some lemmings naturally disperse to other regions in search of better access to food and space. (2) Digging holes–Lemmings are skilled burrowers that use their strong forelimbs and sharp claws to construct intricate underground tunnel systems. These complex burrow networks not only offer shelter from predators and harsh environmental conditions but also serve as storage sites for food and safe spaces for reproduction. (3) Foraging–As herbivores, lemmings primarily consume plant-based resources such as grasses, seeds, fruits, and roots. They forage both above ground and within their burrows, using acute sensory abilities to detect food. During winter, lemmings are even capable of digging beneath snow to access vegetation, and they often stockpile food to survive periods of scarcity. (4) Evading natural predators–Lemmings possess highly developed sensory systems that enable them to detect approaching predators promptly. Upon sensing danger, they emit alarm signals to alert nearby individuals, prompting them to hide or flee to safety. These four core behaviors—migration, digging, foraging, and predator evasion—collectively inspired the development of ALA. This population-inspired metaheuristic algorithm simulates the adaptive and survival strategies of lemmings to enhance global search ability, balance exploration and exploitation, and improve robustness in high-dimensional optimization tasks [33]. The mathematical formulation of ALA during the optimization process is as follows:

(a) Population initialization

As social animals, lemmings search for food and evade predators within a defined spatial range. Accordingly, the initial positions in the optimization process are defined as

(5) $$L_{i}=LB+rand\cdot (UB-LB)$$

where *L_i_* represents the current position of the *i*-th lemming. *LB* and *UB* represent lower and upper boundaries of searching space, respectively. rand is a random number, which is constrained within the range of [0, 1].

(b) Long-distance migration (exploration)

**Figure 2:**
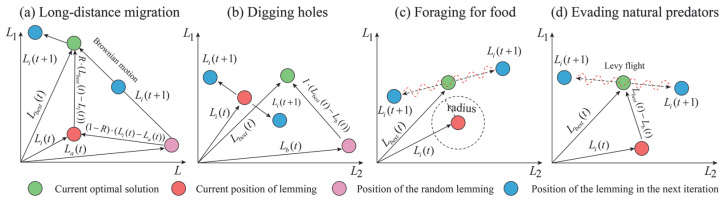
Demonstration of ALA optimization algorithm used in this paper [33].

As food becomes scarce and the population size increases, lemmings must undertake long-distance migration to seek habitats with more abundant resources for the survival of the group. As shown in Figure 2a, this behavior corresponds to the exploration phase of ALA and is mathematically formulated as

(6) $$L_{i}(t+1)=L_{best}(t)+F\cdot BM\cdot (R\cdot (L_{best}(t)-L_{i}(t))+(1-R)\cdot (L_{i}(t)-L_{a}(t)))$$

where $L_{i}(t)$ represents the current position of the *i*-th lemming at the *t*-th iteration. $L_{i}(t+1)$ represents the current position of the *i*-th lemming at the *t* + 1-th iteration. $L_{best}(t)$ represents the current optimal position of the lemming at the *t*-th iteration. $L_{a}(t)$ represents the current position of a random individual lemming at the *t*-th iteration. *F* is a factor that controls the direction of migration. *BM* is a random number, which plays a crucial role in the deep exploration of the search space. *R* represents a factor that controls the movement of the best and random lemmings in the population.

(c) Digging holes (exploration)

Once a suitable habitat is found, lemmings engage in burrowing behavior to prepare for future food acquisition and storage (see Figure 2b). The mathematical expression for this behavior can be formulated as

(7) $$L_{i}(t+1)=L_{i}(t)+F\cdot I\cdot (L_{best}(t)-L_{b}(t))$$

where *I* is a random number. $L_{b}(t)$ represents the current position of a random individual lemming at the *t*-th iteration.

(d) Foraging for food (exploitation)

Within the habitat, lemmings explore the entire space to locate the largest quantity of high-quality food and complete the storage for the group’s survival. As shown in Figure 2c, the position of lemmings during this process can be mathematically represented as

(8) $$L_{i}(t+1)=L_{best}(t)+F\cdot spiral\cdot rand\cdot L_{i}(t)$$

where *spiral* represents the shape of searching space.

(e) Evading natural predators (exploitation)

In the foraging process (see Figure 2d), if lemmings encounter a predator attack, they will quickly execute an avoidance behavior, including retreating to their burrows and utilizing deceptive behaviors to ensure self-protection. The mathematical expression for this behavior can be represented as

(9) $$L_{i}(t+1)=L_{best}(t)+F\cdot G\cdot levy\cdot \left(L_{best}(t)-L_{i}(t)\right)$$

where *G* represents the escape coefficient of lemmings, which decreases with iteration increases. *Levy* is the Levy flight function, which is used to simulate the deceptive behaviors during the escape process.

## 3. Database

Previous studies have identified four key features as primary factors influencing hydrogen storage capacity [33]: surface area, pore volume, pressure, and temperature. Surface area, in particular, is a critical factor affecting the hydrogen storage performance of MOFs. Several techniques are available for determining surface area, among which the Langmuir and Brunauer–Emmett–Teller (BET) methods are the most widely employed. The Langmuir isotherm assumes monolayer adsorption on a homogeneous surface and does not account for multilayer adsorption phenomena [34]. In contrast, the BET approach incorporates the possibility of multilayer adsorption, typically yielding lower surface area values than those derived from the Langmuir model [35]. In this paper, the surface area measured by the BET method was considered as an input feature to predict the hydrogen storage. Furthermore, an increase in pore volume provides additional available space within the MOF structure, enabling it to accommodate a larger number of hydrogen molecules [36]. In addition, pressure and temperature are critical operational parameters for accurately predicting hydrogen storage capacity. For example, physisorption-based adsorbents typically exhibit higher gas storage capacities at cryogenic temperatures. At ambient conditions, weakened van der Waals forces lead to reduced storage capacity [36]. In light of the challenges associated with the experimental determination of hydrogen storage performance of MOFs, this paper utilizes 294 experimental datasets compiled by Salehi et al. [37] to generate the predictive models. In these experiments, hydrogen storage is primarily quantified in two primary forms: total and excess storage capacities. To achieve data consistency and ensure the robustness of the model, total hydrogen storage values were converted into excess hydrogen storage values using Equation (10).

(10) $$N_{excess}=N_{total}-d_{gas}V_{pore}$$

where $N_{total}$ and $N_{excess}$ represent the total and excess hydrogen storage, respectively. $d_{gas}$ and $V_{pore}$ represent the density and pore volume of compressed gas, respectively.

**Figure 3:**
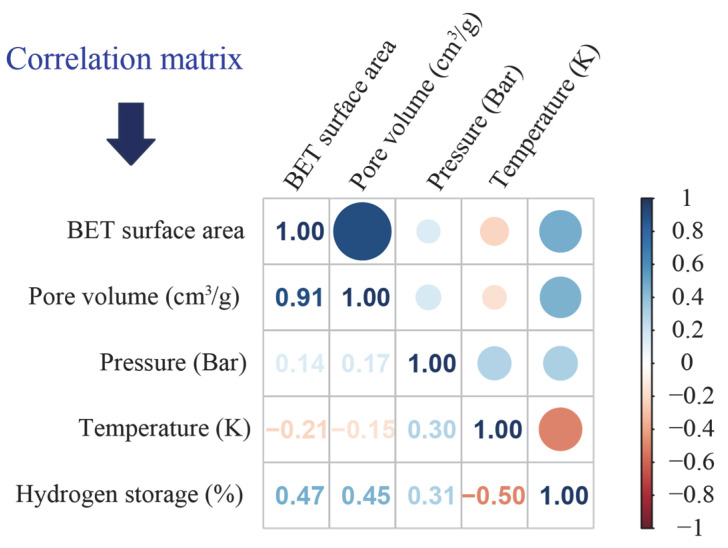
Correlation matrix of all features.

The statistical analyses and correlation test results for all datasets are comprehensively presented in Table 1 and Figure 3, providing a detailed overview of the variables’ statistical properties and the relationships among them. As can be seen from Figure 3, the correlation coefficient between BET surface area and pore volume reaches 0.91, which is attributed to the fact that these two features fundamentally reflect the pore characteristics of MOF materials. For instance, a MOF with a high BET surface area usually possesses a large number of pores with a well-ordered and uniform structure, which in turn results in a high pore volume. In addition, the relatively low correlation coefficients among all input features indicate that the effect of each parameter on the target output is largely independent. On the other hand, the correlation coefficients between most of the input features and the output variables are greater than 0.4. According to the Pearson correlation-based feature selection criterion [38], all input features are deemed suitable for inclusion in the predictive modeling process.

## 4. Development of Prediction Models

**Figure 4:**
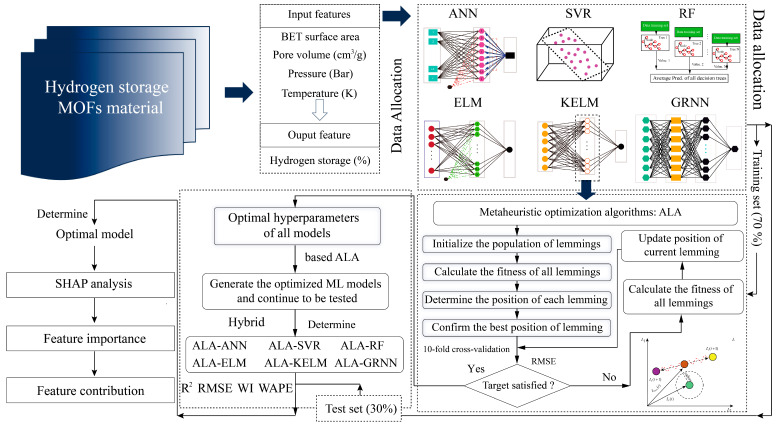
The flowchart of predicting hydrogen storage in MOFs.

In this paper, six ML models were adopted to predict the hydrogen storage performance in MOFs. A novel metaheuristic algorithm (i.e., ALA) was utilized to optimize all models for improving prediction accuracy. To develop high-performance prediction models, as shown in Figure 4, several steps were undertaken:

(1) Data preparation

As previously mentioned, 294 data samples were collected to construct the prediction models. First, the majority of data samples were allocated to form the training set, enabling the models to learn the underlying relationships between input features and the target variable. Subsequently, the remaining samples were assigned to the test set to evaluate the predictive accuracy of the trained models. Common ratios between the training and test sets include 7:3, 8:2, or 9:1. In this paper, a 7:3 ratio (training set: 206 samples and test set: 88 samples) was adopted based on modeling procedures and predictive performance reported in related prior research by Salehi et al. [37]. Furthermore, all input features were normalized into the range of [−1, 1] to prevent overfitting caused by discrepancies in the magnitudes or units of different variables [39].

(2) Model optimization

The predictive accuracy of each model is closely associated with its hyperparameter selection. For the ANN model, the number of hidden layers (*N_h_*) and the number of neurons per layer (*N_n_*) significantly influence model performance, with ranges of [1–3] and [1–10], respectively. For the SVR model, the penalty parameter *C* and the kernel parameter (*k*) are the most influential factors, with ranges of [0.25–252] and [0.25–16], respectively. For the RF model, prediction accuracy is primarily determined by the number of trees (*N_t_*) and the minimum leaf point (*Minleafsize*), varied within the ranges of [1–100] and [1–10], respectively. In the ELM model, selecting an appropriate *N_n_* in the single hidden layer poses a key challenge, with the range defined as [1–150]. The hyperparameter selection and corresponding ranges for the KELM model are consistent with those used in the SVR model. In the GRNN model, the smoothing factor (*S_f_*) is adjusted within the range of 0–5 to evaluate its impact on model performance. Furthermore, the population size and number of iterations significantly influence the optimization performance of the ALA algorithm. Therefore, four values for population sizes (25, 50, 75, and 100) were tested to select the optimal hyperparameters for all ML models over 200 iterations. During this process, a fitness function was constructed to identify the optimal solution for each model. Generally, the root mean square error (RMSE), which does not require consideration of absolute values, is commonly used in conjunction with cross-validation to define the fitness function [40,41]. In this paper, 10-fold cross-validation was combined with RMSE to calculate fitness values for determining the optimal solutions:

(11) $$fitness=\frac{1}{K}\sum_{k=1}^{K}RMSE_{k}$$

where *K* represents the maximum number of subsets in the cross validation.

(3) Model evaluation

After determining the optimal solutions for all models, several indices were utilized to evaluate model performance. In this paper, four statistical indices, including coefficient of determination (R^2^), RMSE, Willmott’s index (WI), and weighted average percentage error (WAPE), were utilized to evaluate model performance. The definitions of these indices are expressed by Equations (12)–(15). Furthermore, several tools such as regression graphs, error analysis, and Taylor graphs were also employed to determine the optimal prediction model.

(12) $$R^{2}=1-\frac{{\left[\sum_{i=1}^{n}\left(Z_{i}-z_{i}\right)\right]}^{2}}{{\left[\sum_{i=1}^{n}\left(Z_{i}-\bar{Z}\right)\right]}^{2}}$$

(13) $$\mathrm{RMSE}=\sqrt{\frac{1}{n}\sum_{i=1}^{n}{\left(Z_{i}-z_{i}\right)}^{2}}$$

(14) $$\mathrm{WI}=1-\left[\frac{\sum_{i=1}^{n}{\left(Z_{i}-z_{i}\right)}^{2}}{\sum_{i=1}^{n}{\left(\left|z_{i}-\bar{Z}\right|+\left|Z_{i}-\bar{Z}\right|\right)}^{2}}\right]$$

(15) $$\mathrm{WAPE}=\frac{{\left[\sum_{i=1}^{n}\left(Z_{i}-z_{i}\right)\right]}^{2}}{{\left[\sum_{i=1}^{n}\left(Z_{i}-\bar{Z}\right)\right]}^{2}}$$

where $Z_{i}$ and $z_{i}$ represent the actual and predicted values of the *i*-th data points in the dataset, respectively. $\bar{Z}$ is the average of actual values. *n* is the maximum number of data points in the dataset.

(4) Model interpretability

To further elucidate the influence of input features on the prediction target, this paper adopts the Shapley additive explanations (SHAP) method to quantify the importance of each input feature and illustrate its contribution to hydrogen storage prediction. This approach improves the interpretability of the “black-box” model used in this paper.

## 5. Results and Discussion

### 5.1. Hyperparameter Selection

**Figure 5:**
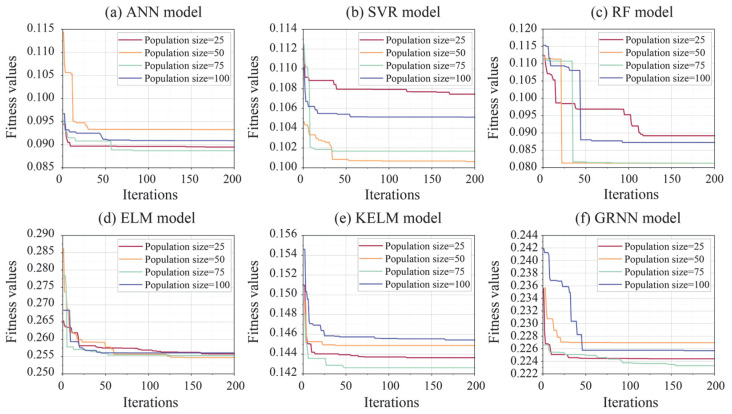
Iteration curves of all models during optimization process.

In this paper, the ALA algorithm was utilized to determine the optimal hyperparameter combinations for each prediction model. Figure 5 demonstrates the fitness curves of all models over 200 iterations. As shown in Figure 5a, all ANN models optimized by ALA with different population sizes reached their minimum fitness values before achieving the maximum number of iterations. Among these models, the ALA-ANN model with a population size of 75 achieved the lowest fitness value. Figure 5b illustrates the iteration results for the optimized SVR models using four different population sizes. Significant variations in fitness values are observed across various population sizes, with the lowest value achieved by the ALA-SVR model at a population size of 50. For the ALA-RF and ALA-ELM hybrid models, changes in population size resulted in only minor differences in minimum fitness values. Notably, the minimum fitness values for hybrid models with 50 and 75 population sizes were nearly identical (see Figure 5c,d). As demonstrated in Figure 5e,f, both ALA-KELM and ALA-GRNN models obtained their minimum fitness values at a population size of 75. Table 2 summarizes the results of fitness values for each model and their optimal hyperparameter combinations after optimization.

### 5.2. Model Performance Evaluation

After obtaining the optimal hyperparameters for all models, the model performance needed to be evaluated to determine the best model for predicting the hydrogen storage. First, the prediction accuracy of the models was verified using the training set. Table 3 presents the results of evaluation indices for all optimized ML models. It can be observed that all models achieved satisfactory prediction performance, as reflected in favorable evaluation indices, with R^2^ values exceeding 0.9. Among these models, the ALA-RF model demonstrated higher competitiveness in hydrogen storage prediction compared to the other models, with higher values of R^2^ and WI (0.9845 and 0.9961) and lower values of RMSE and WAPE (0.2719 and 0.0667). Following this model, ALA-ANN, ALA-SVR, ALA-KELM, and ALA-GRNN, but not the ALA-ELM model, also exhibited strong predictive performance during the training phase.

**Figure 6:**
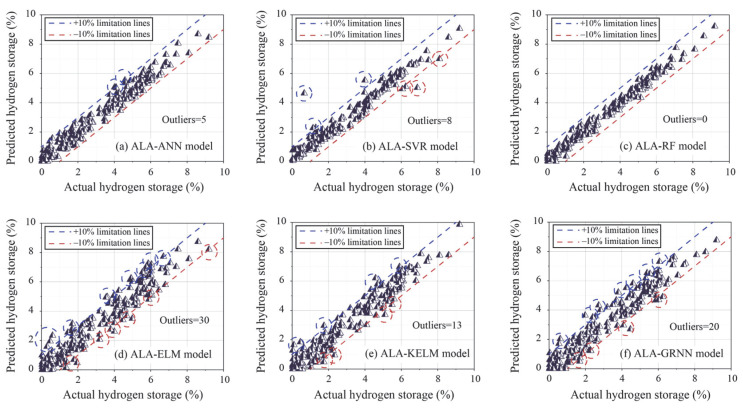
Regression graph of all models based on the training set.

Furthermore, regression graphs were generated to illustrate the prediction performance of all models in the training phase. In a regression graph, the position of each data point is determined by the actual and predicted values. If the predicted values equal actual values, the data points lie on the diagonal. Otherwise, the data points deviate from the diagonal based on the prediction bias. However, perfect predictions are rarely achievable in practical engineering problems. Therefore, a certain degree of prediction error is acceptable. This paper introduces two limitation lines (10%) to assess the predictive accuracy of each model in more detail. Data points falling outside the range of these limitation lines are considered “outliers”. The greater the number of outliers in a model’s predictions, the poorer its performance is deemed to be. As shown in Figure 6, 30 outliers were obtained by the ALA-ELM model, ranking the highest among all models, followed by the ALA-GRNN, ALA-KELM, ALA-SVR, and ALA-ANN models. It is worth noting that the ALA-RF model had zero outliers due to its excellent predictive performance, where all data points were within the boundary line.

However, the performance of a model during the training phase does not necessarily reflect its ultimate applicability in real-world engineering scenarios [42,43]. Therefore, the test set was used to assess the actual predictive performance of the trained models. Table 4 presents the results of evaluation indices for all optimized ML models in the testing phase. It is evident that two models (ALA-ELM and ALA-GRNN) obtained unsatisfactory prediction performance, as indicated by poor evaluation indices, including R^2^ values lower than 0.9. Among the other models, the ALA-RF model demonstrated the strongest performance in predicting hydrogen storage, achieving the highest values of R^2^ and WI (0.9840 and 0.9959) and the lowest values of RMSE and WAPE (0.2828 and 0.0714). Following ALA-RF, the ALA-ANN, ALA-SVR, and ALA-KELM also exhibited strong predictive performance during the testing phase.

**Figure 7:**
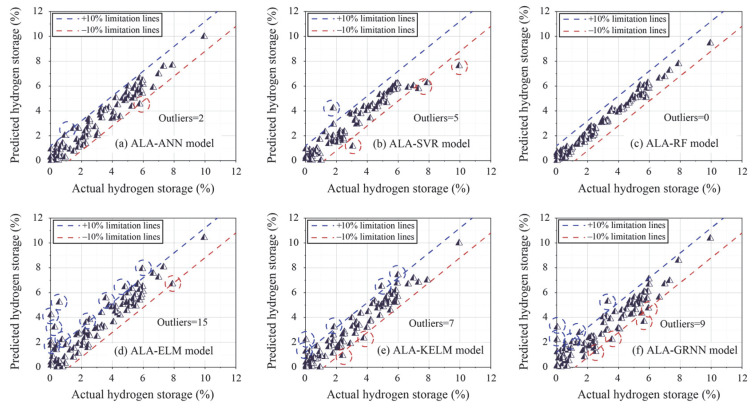
Regression graphs of all models based on the test set.

Figure 7 illustrates the regression graphs of all models in the testing phase. As shown in this figure, ALA-ELM and ALA-RF represent the worst and best-performing models among all models, respectively. For ALA-ELM, 15 outliers are distributed outside the limitation lines, whereas ALA-RF shows no outliers in its prediction results. Following ALA-RF, the number of outliers obtained with the ALA-GRNN, ALA-KELM, ALA-SVR, and ALA-ANN models are 9, 7, 5, and 2, respectively.

**Figure 8:**
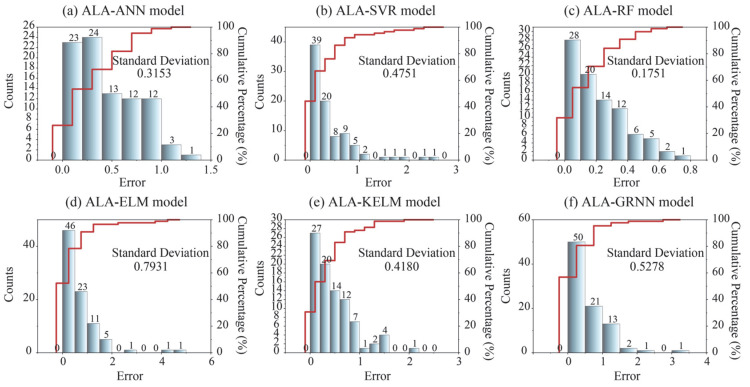
Error distribution of all models for predicting hydrogen storage in the testing phase.

Moreover, error analysis was conducted to further compare model performance using the test set. Figure 8 shows the error distribution between actual and predicted values for each model. It is evident that all models exhibit a high proportion of prediction errors less than 1, with only the ALA-RF model maintaining prediction errors within 1 for all test samples. Among these models, both ALA-RF and ALA-ANN display a more uniform cumulative distribution of errors and lower maximum error values compared to the others. In contrast, ALA-ELM and ALA-GRNN demonstrate significantly more instances of large prediction errors. Furthermore, this paper employs the standard deviation of prediction errors as a metric to rank the models’ predictive accuracy. A lower standard deviation indicates that the prediction errors are more concentrated, stable, and less volatile. When considered alongside the error distribution analysis, this metric enables a comprehensive evaluation of model performance [44]. The results indicate that the minimum standard deviation of prediction errors was obtained by the ALA-RF model (0.1751), and the ALA-ELM model obtained the maximum standard deviation of prediction errors (0.7931). Notably, ALA-KELM’s prediction error standard deviation was lower than that of ALA-SVR. This outcome may be attributed to large prediction deviations for certain individual samples in the ALA-SVR model.

**Figure 9:**
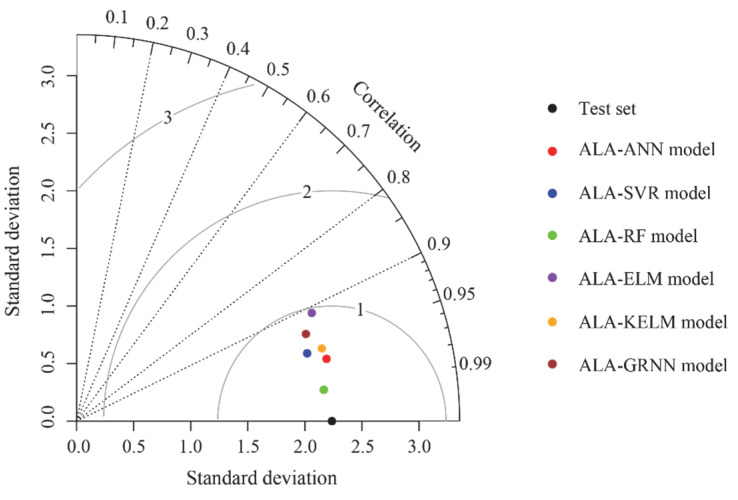
Taylor graph of all models based on the test set.

Therefore, it was necessary to further validate the performance of each model. In this paper, a Taylor graph tool was employed to determine the final performance of all models. In a Taylor graph, the position of each model is determined by three indices, including standard deviation, RMSE, and correlation coefficient. For the test set point, the reference point has a correlation coefficient of 1 and an RMSE of 0, as it lies on the horizontal axis quantified by the standard deviation. For other models developed in this paper, the position relative to the test points reflects their predictive performance. In principle, a model that is closer to the test point indicates better performance. As shown in Figure 9, the ALA-RF model was closest to the test point, followed by the ALA-ANN model. Notably, the ALA-SVR model was closer to the test point compared to the ALA-KELM model, suggesting that the former demonstrated superior predictive performance.

Additionally, a previous study [37] revealed that the committee machine intelligence system (CMIS) model obtained high performance in predicting hydrogen storage, with an overall R^2^ of 0.982. To further compare the performance of various models in hydrogen storage prediction, three ALA-RF models were reconstructed under different data splitting ratios (6:4, 8:2, and 9:1). As shown in Table 5, the results of performance evaluations indicated that model accuracy on the training set improved as the number of training samples increased. However, when the training set accounted for more than 70% of the total data, the performance gain became marginal, suggesting diminishing returns with additional training data. Based on the R^2^ metric, the ALA-RF obtained higher prediction accuracy in both the training (R^2^ of 0.9845) and testing (R^2^ of 0.9840) phases based on the same data allocation (training set: test set = 7:3). In conclusion, the ALA-RF model was the best-performing model for predicting hydrogen storage among the developed models.

### 5.3. Model Interpretability

**Figure 10:**
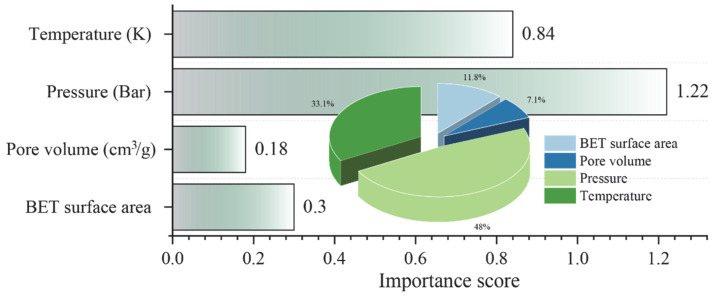
Importance score of all features in predicting hydrogen storage.

**Figure 11:**
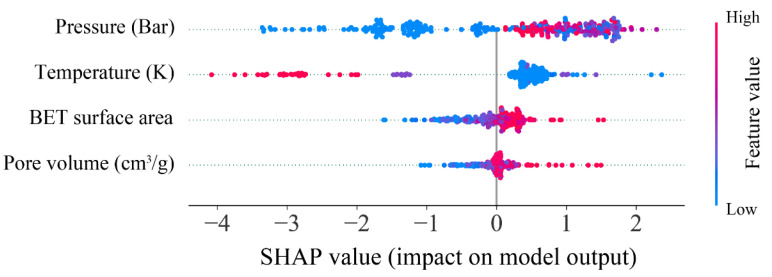
SHAP values of all features in predicting hydrogen storage.

After identifying the optimal model for predicting hydrogen storage in MOFs, the impact and contribution of the input features to the prediction target were analyzed using the SHAP method. Figure 10 shows the importance score of all features in predicting hydrogen storage through the SHAP analysis. Pressure was found to have the greatest impact on the prediction of hydrogen storage, with the highest importance score of 1.22. This was followed by temperature (0.84), BET surface area (0.30), and pore volume (0.18). Furthermore, the contribution of each feature to hydrogen storage prediction is illustrated in Figure 11. It is evident that pressure, BET surface, and pore volume positively influence the prediction of hydrogen storage, indicating that increases in these features correspond to increases in the predicted values of hydrogen storage. With respect to temperature, a negative correlation with hydrogen storage was observed, suggesting that reducing temperature contributes to an increase in hydrogen storage. These findings are consistent with those reported by Salehi et al. [37], thereby reinforcing the validity of the current analysis.

## 6. Conclusions

In this paper, six ML models were adopted to predict hydrogen storage in MOFs based on 294 data samples. A novel metaheuristic optimization algorithm, ALA, was employed to further improve model performance. After evaluating the model performance, SHAP analysis was conducted to calculate the feature importance score and impact on hydrogen storage prediction. The main conclusions are summarized as follows:

(1) The evaluation results demonstrated that the ALA-RF model was the optimal model for predicting hydrogen storage in MOFs, yielding the most satisfactory performance in both the training and testing phases. The values of R^2^, RMSE, WI, and WAPE were 0.9845, 0.2719, 0.9961, and 0.0667 (training set) and 0.9840, 0.2828, 0.9959, and 0.0714 (test set), respectively. In prediction accuracy, the model provided by this paper outperformed the previous model developed using the same database.
(2) According to the SHAP analysis, pressure was identified as the most important feature for predicting hydrogen storage in MOFs, with the highest importance score of 1.22 among all input features. Temperature exhibited the most significant negative contribution to the prediction results for hydrogen storage.

However, the proposed model demonstrated strong predictive performance, which was limited by the relatively small and structurally narrow dataset of 294 MOF samples. These factors may restrict the model’s generalizability to unexplored or more complex MOF chemistries. The absence of molecular-level descriptors—such as specific metal nodes, organic linkers, or functional groups—also limits the ability to capture important steric and electronic effects that influence hydrogen adsorption. Furthermore, although pressure and temperature were identified as critical factors, the current work did not extend these insights to virtual screening or structural optimization. Future efforts should integrate machine learning models with high-throughput molecular simulations to explore a broader design space and guide the rational development of novel MOFs. Additionally, developing an automated recommendation system that connects predictive models with operational constraints could enable inverse design and accelerate experimental validation.

## Figures and Tables

**Table 1:** Statistical analyses of all features with the database.

Features	Unit	Statistical Indices			
		Median	Standard Deviation	Minimum	Maximum
BET surface area	/	2798.50	1225.16	150.00	6240.00
Pore volume	cm^3^/g	1.19	0.52	0.04	3.60
Pressure	Bar	18.24	36.51	0.19	100.00
Temperature	K	77.00	74.10	30.00	300.00
Hydrogen storage	%	2.75	2.21	0.03	9.95

**Table 2:** Fitness values and optimal hyperparameter combinations of all models.

Models	Population Sizes				Hyperparameters
	25	50	75	100	
ALA-ANN	0.08949	0.09328	0.08869	0.09088	*N_h_*: 1; *N_n_*: 8
ALA-SVR	0.10745	0.10063	0.10168	0.10512	*C*: 135.2; *k*: 1.35
ALA-RF	0.08920	0.08122	0.08124	0.08724	*N_t_*: 45; *Minleafsize*: 1
ALA-ELM	0.25577	0.25471	0.25532	0.25606	*N_n_*: 72
ALA-KELM	0.14364	0.14487	0.14263	0.14543	*C*: 114.5; *k*: 1.87
ALA-GRNN	0.22441	0.22696	0.22330	0.22568	*S_f_*: 0.4

**Table 3:** Statistical results of four evaluation indices for six models in training phase.

Models	Evaluation Indices			
	R^2^	RMSE	WI	WAPE
ALA-ANN	0.9649	0.4098	0.9909	0.1031
ALA-SVR	0.9411	0.5306	0.9848	0.0922
ALA-RF	0.9845	0.2719	0.9961	0.0667
ALA-ELM	0.9063	0.6693	0.9769	0.1697
ALA-KELM	0.9327	0.5672	0.9834	0.1420
ALA-GRNN	0.9232	0.6058	0.9808	0.1500

**Table 4:** Statistical results of four evaluation indices for six models in testing phase.

Models	Evaluation Indices			
	R^2^	RMSE	WI	WAPE
ALA-ANN	0.9649	0.4098	0.9909	0.1031
ALA-SVR	0.9411	0.5306	0.9848	0.0922
ALA-RF	0.9845	0.2719	0.9961	0.0667
ALA-ELM	0.9063	0.6693	0.9769	0.1697
ALA-KELM	0.9327	0.5672	0.9834	0.1420
ALA-GRNN	0.9232	0.6058	0.9808	0.1500

**Table 5:** Performance comparison between the previous and present models.

Models	Ratio of Training Set to Test Set	R^2^	
		Training	Test
ALA-RF	6:4	0.9705	0.8917
ALA-RF	7:3	0.9845	0.9840
ALA-RF	8:2	0.9875	0.9421
ALA-RF	9:1	0.9892	0.9213
CMIS	7:3	0.9830	0.9780

## Data Availability

The original contributions presented in the study are included in the article, further inquiries can be directed to the corresponding authors.
